# PEG-Induced Osmotic Stress Alters Root Morphology and Root Hair Traits in Wheat Genotypes

**DOI:** 10.3390/plants10061042

**Published:** 2021-05-21

**Authors:** Arif Hasan Khan Robin, Shatabdi Ghosh, Md. Abu Shahed

**Affiliations:** Department of Genetics and Plant Breeding, Bangladesh Agricultural University, Mymensingh 2202, Bangladesh; shatabdi41465@bau.edu.bd (S.G.); shahed41483@bau.edu.bd (M.A.S.)

**Keywords:** wheat, PEG, osmotic stress, root traits, principal component analysis

## Abstract

Wheat crop in drought-prone regions of Bangladesh suffers from osmotic stress. The objective of this study was to investigate the response of wheat genotypes with respect to root morphology and root hair traits under polyethylene glycol (PEG)-induced osmotic stress. A total of 22 genotypes of wheat were grown hydroponically and two treatments—0% and 10% PEG—were imposed at 14 days after germination. Plant growth was reduced in terms of plant height, number of live leaves per tiller, shoot dry weight, number of root-bearing phytomers, and roots per tiller. Notably, PEG-induced osmotic stress increased root dry weight per tiller by increasing length of the main axis and lateral roots, as well as the diameter and density of both lateral roots and root hairs of the individual roots. A biplot was drawn after a principal component analysis, taking three less-affected (high-yielding genotypes) and three highly affected (low-yielding genotypes and landrace) genotypes under 10% PEG stress, compared to control. Principal component 1 separated PEG-treated wheat genotypes from control-treated genotypes, with a high and positive coefficient for the density of lateral roots and root hairs, length and diameter of the main axis, and first-order lateral roots and leaf injury scores, indicating that these traits are associated with osmotic stress tolerance. Principal component 2 separated high-yielding and tolerant wheat genotypes from low-yielding and susceptible genotypes, with a high coefficient for root dry weight, density of root hairs and second-order lateral roots, length of the main axis, and first-order lateral roots. An increase in root dry weight in PEG-stress-tolerant wheat genotypes was achieved through an increase in length and diameter of the main axis and lateral roots. The information derived from this research could be exploited for identifying osmotic stress-tolerant QTL and for developing abiotic-tolerant cultivars of wheat.

## 1. Introduction 

Wheat (*Triticum aestivum* L.) is one of the most commonly adapted cereal plants in different growing environments worldwide. It is the second of the world’s top three primary cereals, with 730.5 million tons of global annual production from 215.2 million hectares of land in 2018–2019 [1]. Limited water availability is a major issue for wheat production around the world [2]. Approximately 45% of the wheat-growing lands in developing countries are vulnerable to drought [3]. The situation of wheat production may become more problematic since the area affected by the drought stress may increase four times by the middle of the 21st century [4]. Drought stress significantly reduces cereal crop production globally by 10 percent due to its negative effects on plant growth and grain productivity [4]. The best option to mediate the situation is to develop drought-tolerant crop varieties. In this regard, developing wheat varieties with efficient root systems that can exploit residual soil moisture under water-deficient conditions in the dry season is important.

**Figure 1:**
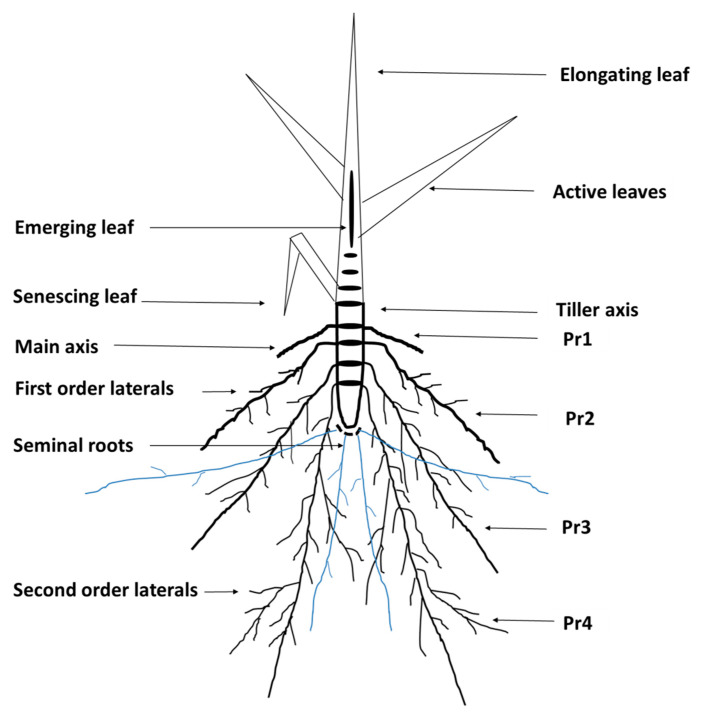
Developmental stages of leaf and root-bearing phytomers (Pr) at the tiller axis of wheat plant in a stylized diagram. Pr, root-bearing phytomer. Blue lines represent seminal roots.

Research on drought tolerance still has to deal with many underexplored aspects associated with root traits. Understanding the root systems of plants at the phytomer level may help to dissect the complicated root architecture of roots. Phytomer is known to be the vegetative unit consisting of a stem, the proximal internodes, an axillary bud and its subtending branch, and primordia of nodal roots (Figure 1) [5]. Thus, phytomers are the recurring structural units in the tiller axis of cereals and grasses that originated from the apical meristem and are considered as the building blocks of a tiller [6,7,8]. Phytomer-level data can explicitly describe the intrinsic root development of poaceous plants, from newly formed roots to their final size, which provides a better understanding of root responses toward stressed environments [9]. In the root-bearing phytomers, the elongation of the main root axis starts at the first root-bearing phytomer (Pr1), and the first-order lateral (FOL) and second-order lateral (SOL) roots form as the roots get older at the successively older phytomers (Figure 1). 

As a genotype-specific response, osmotic stress leads to physiological dysfunction caused by the sudden change in the solute concentration around a cell, affecting morphological traits including root length, dry weight, and root–shoot ratio [10]. It was reported that osmotic stress conditions reduced root growth and root apical meristem (RAM) size, promoting premature cell differentiation without affecting the stem cell niche morphology [11]. Osmotic stress represses germination percentage, seed vigor index, length of coleoptile, length of shoot and root, osmotic membrane stability, and consequently, suppresses plant growth [12]. It is generally believed that a deep, widespread, and branched root system is essential for developing drought-tolerant crops [13]. Therefore, a clear understanding of root system architecture, morphology of root traits, and root hairs in response to osmotic stress, induced by the drought stress, is required for future wheat improvement programs. 

Root traits in wheat are the sensitive plant characteristics under drought stress [14,15,16]. As an initial response, PEG-induced osmotic stress extends the root tips of the main axes by expanding the root apical meristem [16]. In addition, the traits of root hairs that are influenced by the number of soil, rhizosphere-related, nutritional, and microbial factors are also sensitive in wheat and barley [17,18,19]. In the last 20 years, knowledge on root morphology of annual crop plants, such as wheat, barley, or perennial forage grass under a limited supply of water, has developed [16,20,21]. Large root systems in wheat absorb more water and nitrogen in dry environments to promote access to sufficient water for grain filling [22].

Polyethylene glycol is a commonly used compound in a hydroponic culture that creates lower osmotic potential [23]. In a previous study, PEG-induced mild osmotic stress reduced the length, density, and diameter of root hairs [24]. In castor bean, PEG-induced osmotic stress repressed the growth of plants by reducing total root length, surface area, root volume, and the number of root tips and thus affected cadmium accumulation [25]. Genotype-specific effects on root morphology and root hair traits were also observed under PEG-induced osmotic stress at the vegetative growth stage of rice [26,27], wheat [28,29], and other cereal crop species [30]. Therefore, the study was designed to explore the impact of osmotic stress induced by 10% PEG-6000 during the growth and development of seminal and adventitious roots at the phytomer level [16,31,32]. This experimentation involved measuring plastic alterations of root morphology and root hair traits at the phytomer level (Figure 1) [9,33,34]. Thus, the objective of this study was to identify the root traits and root hair traits responsive to osmotic stress in tolerant versus susceptible wheat genotypes in PEG-treated hydroponic culture.

## 2. Results

### 2.1. Effects of PEG Treatment 

**Figure 2:**
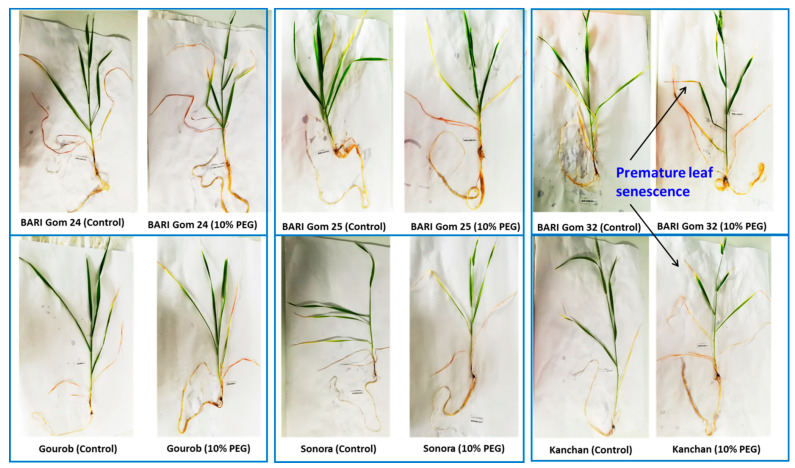
Effect of 10% PEG-induced osmotic stress on wheat genotypes, compared to control. Osmotic stress caused premature senescence of leaves in wheat genotypes.

**Figure 3:**
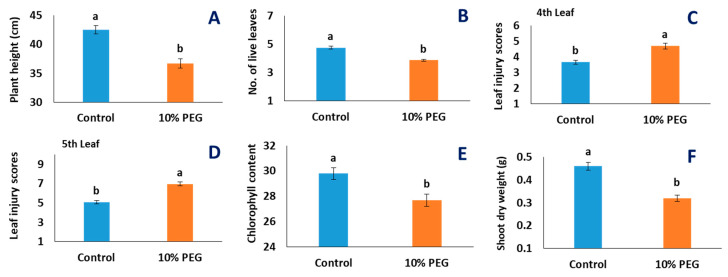
Effects of PEG-induced osmotic stress on morphological traits of shoot of 22 wheat genotypes. Each data bar represents the average of 3 replicates from 22 genotypes: (**A**) plant height (cm); (**B**) total number of live leaves; (**C**) leaf injury scores for the fourth leaf; (**D**) leaf injury scores for the fifth leaf; (**E**) chlorophyll content; (**F**) shoot dry weight in g. The statistical significance was tested following a general linear model and a post hoc analysis was conducted following Tukey’s pairwise comparison. Letters ‘a’ and ‘b’ denote significant differences. Vertical bars indicate standard error of means.

PEG-induced osmotic stress in the wheat plants significantly altered the shoot, root, and root hair traits (Table 1, Figure 2). As an initial symptom, PEG-induced osmotic stress caused premature leaf senescence by degrading chlorophyll of the active leaf tissues starting from the tips downwards (Figure 2). Plant height, number of live leaves per tiller, chlorophyll content, and shoot dry weight per tiller were decreased upon 10% PEG treatment over control (Figure 3, Table 1). PEG-induced osmotic stress reduced plant height by 14% and shoot dry weight by 30%, indicating that both elongations of leaves and dry matter deposition were affected (Figure 3). 

**Figure 4:**
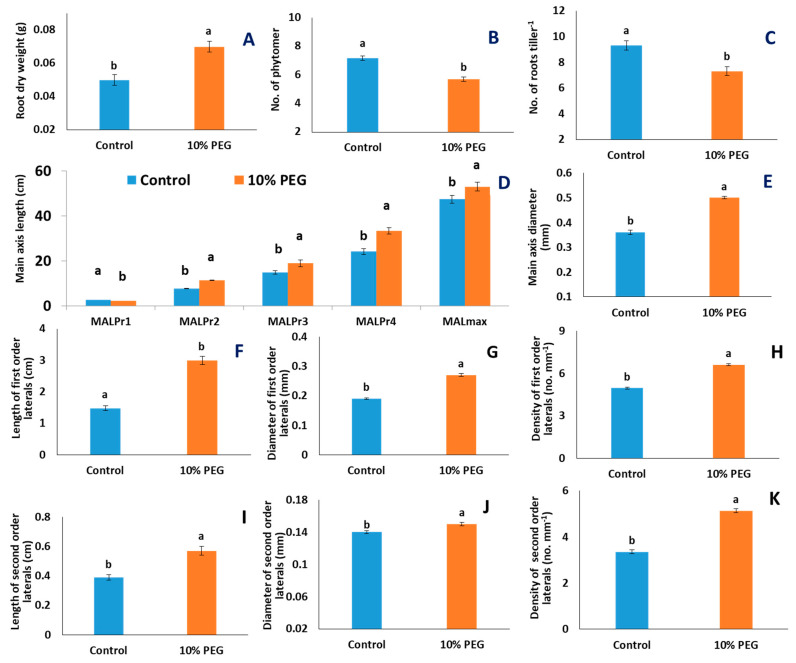
Effects of PEG-induced osmotic stress on morphological traits of roots of 22 wheat genotypes. Each data bar in A, B–E, and F–L represents the average of 3, 2, and 10 replicates from 22 genotypes, respectively: (**A**) root dry weight (g); (**B**) number of root-bearing phytomers per tiller; (**C**) number of roots per tiller; (**D**) main axis length (MAL) at the root-bearing phytomer (Pr1–4) and maximum main axis length (MALmax); (**E**) main axis diameter; (**F**–**H**) length (cm), diameter (mm), and density (no. mm^−1^) of first-order lateral roots; (**I**–**K**) length (cm), diameter (mm), and density of second-order lateral roots (no. mm^−1^). The statistical significance was tested following a general linear model and a post hoc analysis was conducted following Tukey’s pairwise comparison. Letters ‘a’ and ‘b’ denote significant differences. Vertical bars indicate standard error of means.

**Figure 5:**
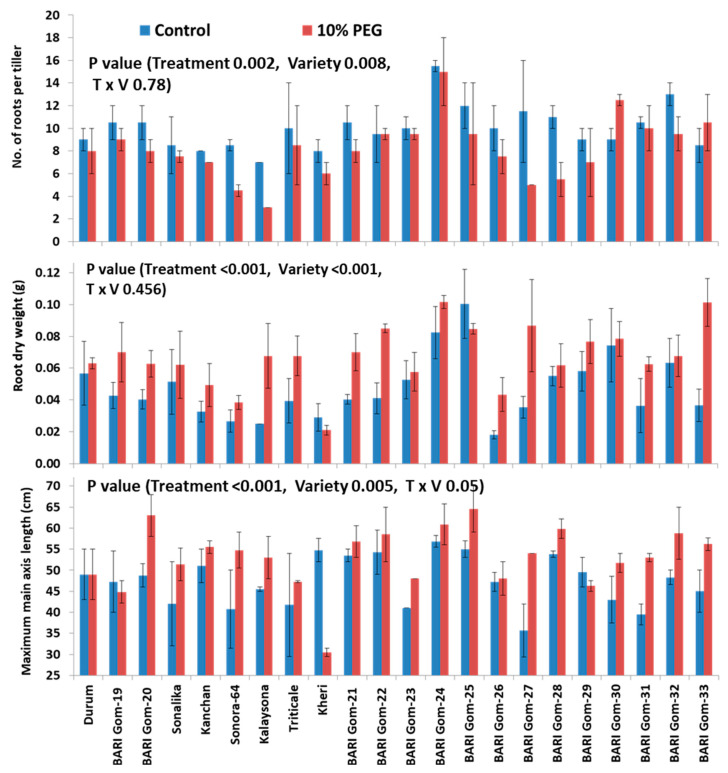
Effects of PEG-induced osmotic stress on selected root traits of wheat genotypes. Each data bar represents average of three replicates for root dry weight and that of two replicates for number of roots per tiller and maximum main axis length. Vertical bars represent standard error of mean. The statistical significance was tested following a two-way ANOVA under a general linear model procedure.

PEG-induced hydroponic culture reduced the number of root-bearing phytomers per tiller by 20.1% (Figure 4B) and the total number of roots per tiller by 21.6% (Figure 4C and Figure 5), but root dry weight per tiller increased by 40% (*p* < 0.001, Figure 4A and Figure 5). The increase in root dry weight under 10% PEG treatment coincided with an increase in the main axes length in root-bearing phytomers 2, 3, and 4 (Pr2–Pr4), and in maximum main axis length by 11.7%, as well as increased values for main axis diameter, length, diameter, and density of both first- and second-order lateral roots (Figure 4). 

**Figure 6:**
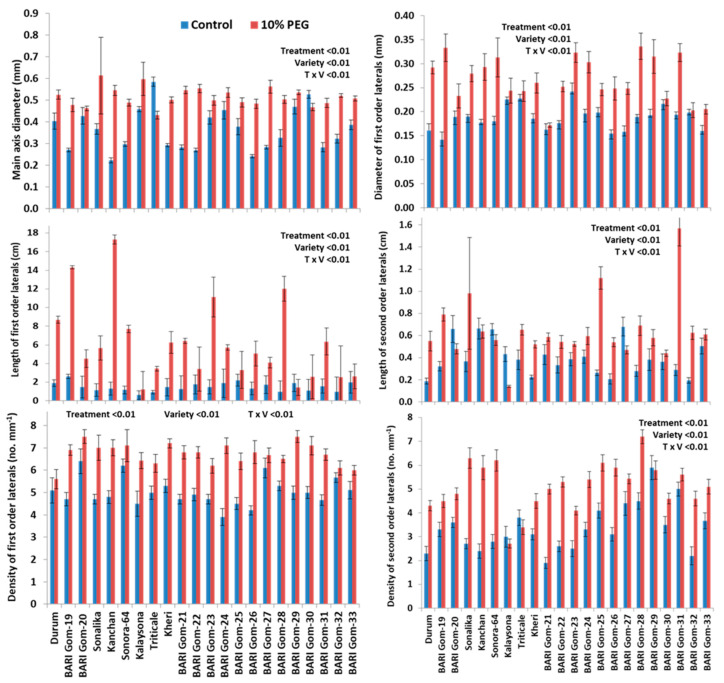
Effects of PEG-induced osmotic stress on diameter of main axis and lateral root traits of 22 wheat genotypes. Each data bar represents average of 10 replicates. Vertical bars represent standard error of mean. The statistical significance was tested following a two-way ANOVA under a general linear model procedure.

The length of main axis at Pr2, Pr3, Pr4 and maximum were increased by 49.7%, 27.2%, 38.2%, and 11.7%, respectively (*p* < 0.001, Figure 4D and Figure 5); diameter of main axis was increased by 38.9% (*p* < 0.001, Figure 4E and Figure 6), but the main axis length at the youngest Pr was decreased by 13% under PEG treatment, compared to control (Figure 4D and Figure 6). Length, diameter, and density of first-order lateral roots significantly were increased by 102.7% (*p* < 0.001, Figure 4F and Figure 6), 42.1% (*p* < 0.001, Figure 4G and Figure 6), and 33.3% (*p* < 0.001, Figure 4H and Figure 6), respectively, under PEG-treated condition, compared to control. Length, diameter, and density of second-order lateral roots were also significantly increased by 46.2% (*p* < 0.001, Figure 4I and Figure 6), 7.1% (*p* < 0.01, Figure 4J), and 53.1% (*p* < 0.001, Figure 4K and Figure 6), respectively, under PEG-treated condition, compared to control. 

**Figure 7:**
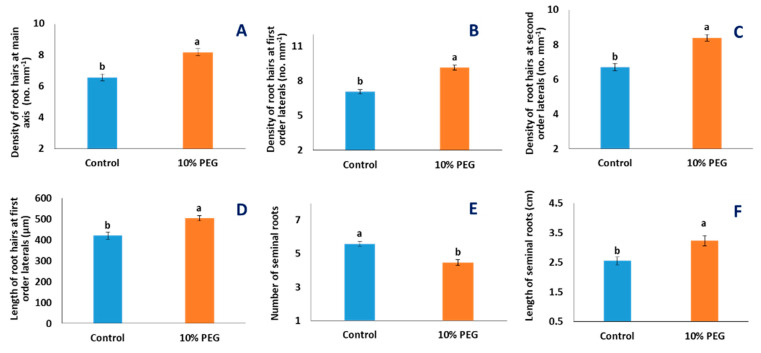
Effects of PEG-induced osmotic stress on root hairs and seminal roots of 22 wheat genotypes. Each data bar in A–D, E, and F represents the average of 5, 2, and 10 replicates from 22 genotypes, respectively: (**A**–**C**) density of root hairs of main axis, first-order, and second-order lateral roots; (**D**) length of second-order lateral roots; (**E**) number of seminal roots; and (**F**) length of seminal roots. The statistical significance was tested following a general linear model and a post hoc analysis was conducted following Tukey’s pairwise comparison. Letters ‘a’ and ‘b’ denote significant differences. Vertical bars indicate standard error of means.

**Figure 8:**
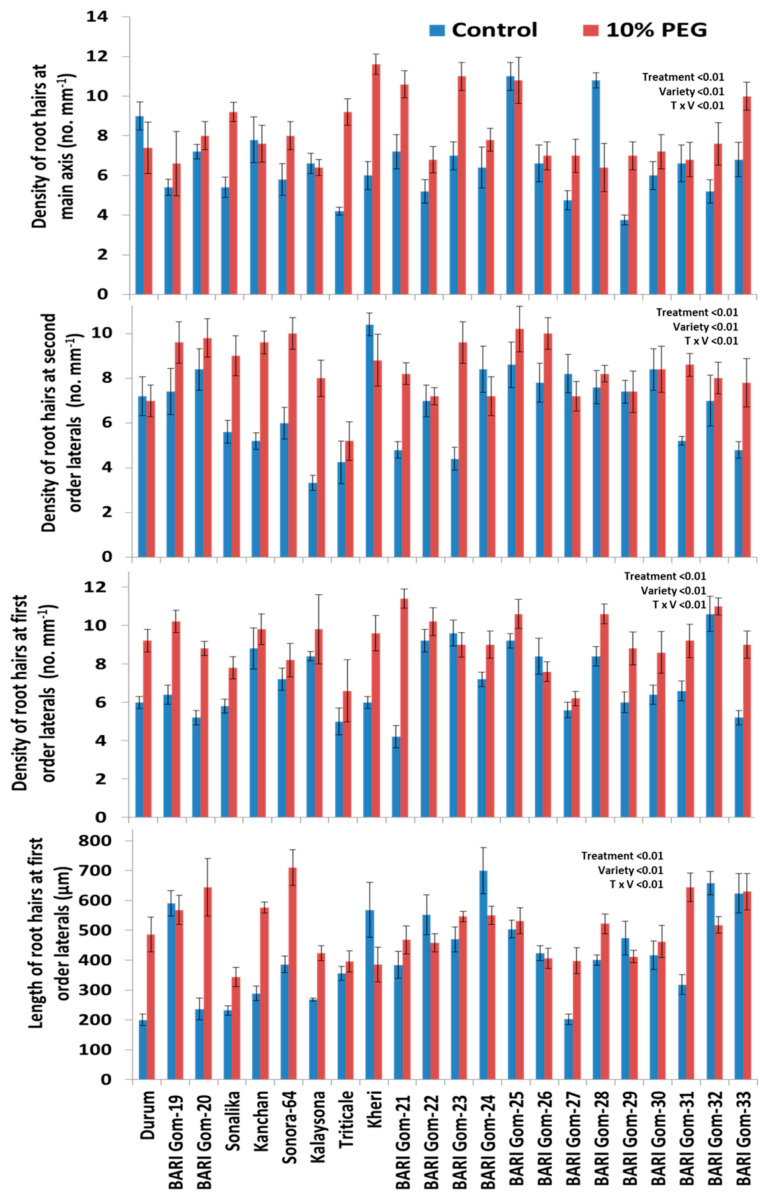
Effects of PEG-induced osmotic stress on root hairs of 22 wheat genotypes. Each data bar represents the average of five replicates from 22 genotypes, respectively. The statistical significance was tested following a two-way ANOVA under a general linear model procedure. Vertical bars indicate standard error of means.

Root hair density on the main axis was increased by 24.3% (*p* < 0.001, Figure 7A) under PEG treatment, the density of root hairs on first-order lateral roots was increased by 29.6% (*p* < 0.001, Figure 7B and Figure 8), and the density of root hairs on second-order lateral roots was increased by 25.5% (*p* < 0.001, Figure 7C and Figure 8) under PEG treatment. Length of root hairs on first-order lateral roots was increased by 19.8% (*p* < 0.001, Figure 7D and Figure 8) under PEG treatment, compared to the control condition. The number of seminal roots was significantly reduced by 19.9% (*p* < 0.001, Figure 7E) in the treated condition, but the length of seminal roots was increased by 26.7% (*p* < 0.001, Figure 7F and Figure 8) in PEG-treated condition, compared to the control condition.

### 2.2. Varietal Differences

A significant difference was observed in shoot, root, and root hair traits of 22 wheat genotypes except for the total number of phytomers (Table S1). The genotype BARI Gom-24 recorded the highest root dry weight (0.10 g, Figure 5), the total number of root-bearing phytomers (7.75), total roots per phytomer (12.25, Figure 5), and the main axis length at phytomer 1 (3.05 cm). The genotype BARI Gom-25 recorded the highest main axis length (59.75 cm, Figure 5), the density of root hairs on the main axis (10.9 roots per mm, Figure 8), and the number of seminal roots (6.25) (Table S1). This genotype also recorded the second-highest root dry weight (0.09 g) per tiller (Figure 5). In contrast, the low-yielding genotype Kalaysona recorded the lowest plant height (32 cm), the total number of roots per tiller (5.0, Figure 5), length of second-order lateral roots (0.29 cm, Figure 6), and the density of second-order lateral roots (0.13 roots per mm, Figure 6). Another low-yielding genotype, Sonora, recorded the lowest total number of live leaves (3.67 per tiller) and shoot dry weight (0.34 g per tiller). Both Kalaysona and Sonora recorded the lowest total number of root-bearing phytomers (5.5) (Table S1).

### 2.3. Treatment–Genotype Interactions 

This study investigated the response of 22 diverse wheat genotypes under PEG-induced osmotic stress with respect to the morphology of roots and root hairs. Despite the significant effects of treatment of PEG-induced osmotic stress on root morphology, all genotypes did not respond in a similar fashion. A number of wheat genotypes showed significant treatment–genotype interactions. Length of seminal roots was increased in most genotypes under PEG treatment but was decreased in the genotype Sourav (8.3%), Kalaysona (22.9%), BARI Gom-21 (21.4%), BARI Gom-24 (14.8%), BARI Gom-27 (68%), and BARI Gom-29 (15.8%) under PEG treatment, compared to control (*p* < 0.01, Table S2). 

Main axis length at phytomer 1 was increased in the genotype Durum (55.6%), Sonalika (67.7%), Kalaysona (10%), BARI Gom-27 (84.2%), BARI Gom-28 (22%), BARI Gom-29 (47.4%), and BARI Gom-31 (46.9%) but was decreased in the rest of the genotypes under PEG treatment, compared to control (*p* < 0.001, Table S2). The maximum main axis length was increased in most of the genotypes but was reduced in Kheri (44.3%), BARI Gom-29 (6.5%), and BARI Gom-29 (5%) under PEG treatment, compared to control (*p* < 0.05, Figure 5). The main axis length was also increased in Pr2, Pr3, and Pr4 in the majority of the wheat genotypes (Table S2). The main axis diameter was increased in most of the genotypes under PEG stress but was reduced in Triticale (26%) and BARI Gom-27 (11%) under PEG treatment, compared to control (*p* < 0.001, Figure 6).

Significant treatment–genotype interaction was also noted in lateral root traits. Length, diameter, and density of first-order lateral roots were significantly increased in all genotypes except for the length of first-order lateral roots in the genotype BARI Gom-29 (22%) under PEG treatment, compared to control (*p* < 0.001, Figure 6). Strikingly, length, diameter, and density of second-order lateral roots were significantly increased in majority genotypes except for the length of second-order lateral roots in the genotype Kalaysona (67%) and Gourab (40%) under PEG treatment, compared to control (*p* < 0.001, Figure 6). The diameter of first-order lateral roots was increased in the majority of the genotypes, with a higher increase in BARI Gom-19 and BARI Gom-28 under PEG treatment, compared to control (*p* < 0.001, Figure 6). Similarly, the density of second-order lateral roots was increased in all genotypes, with the highest increase in BARI Gom-24 under PEG stress (*p* < 0.001, Figure 6). 

Among the root hair traits, the root hair density on the main axis was increased in the majority of the genotypes but was reduced in BARI Gom-28 by 40.7% under PEG treatment, compared to control (*p* < 0.001, Figure 8). The density of root hairs of first-order lateral roots was increased in all genotypes but was reduced in BARI Gom-26 (9.5%) under PEG treatment, compared to control (*p* < 0.001, Figure 8). Thus, the genotype-specific responses of wheat genotypes toward the osmotic stress enable wheat breeders to select suitable wheat genotypes to mitigate drought stress.

### 2.4. Trait Association

A number of root traits responded similarly under PEG-induced osmotic stress, but all root traits were not strongly correlated to each other due to genotypic variations (Table S3). Correlation study revealed that, notably, the number of seminal roots per tiller was positively correlated with the total number of nodal roots per tiller, and root dry weight per tiller was positively associated with the maximum main axis length of roots (Table S3). 

**Figure 9:**
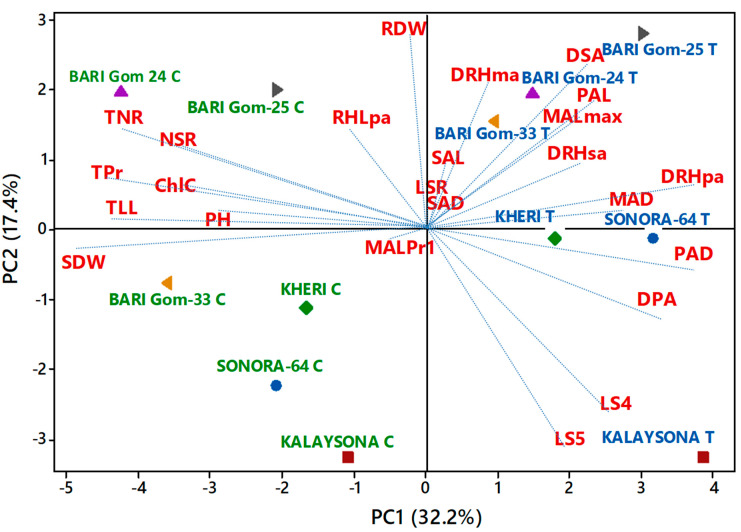
Biplot of root morphology and root hair traits of six wheat genotypes under two treatments (C, control, and T, 10% PEG). Data points indicate PC scores of each wheat genotype under each treatment: PH = plant height; TLL = total no. of live leaves; LS4 = leaf injury scores for the fourth leaf; LS5 = leaf injury scores for the fifth leaf; ChlC = chlorophyll content; SDW = shoot dry weight; RDW = root dry weight; TPr = total number of root-bearing phytomers per tiller; TNR = total number of roots per tiller; NSR = number of seminal roots; LSR = length of seminal roots; MALmax = maximum main axis length; MALPr1 = main root axis length at phytomer 1; MAD = main axis diameter; PAL = length of first-order lateral roots; PAD = diameter of first-order lateral roots; DPA = density of first-order lateral roots; SAL = length of second-order lateral roots; SAD = diameter of second-order lateral roots; DSA = density of second-order lateral roots; DRHMA = density of root hairs of main axis; DRHSA = density of root hairs of second-order lateral roots; DRHPA = density of root hairs of first-order lateral roots; RHLPA = length of root hairs of first-order lateral roots.

The most apposite association among the traits under the study was obtained from the principal component analysis (PCA), in which the vector length on the biplot exhibited the magnitude of variation explained by the respective traits and genotype–treatment combinations (Figure 9). The first four principal components (PC) explained 70.7% of the total data variation for the effect of PEG stress on some important root and root hair traits. PC1, PC2, PC3, and PC4 explained 32.2%, 17.4%, 11.9%, and 9.2% data variation, respectively (Table 2). The eigenvalues of the first four PCs were greater than unity. Nevertheless, the first two PCs exhibited 50% of the total variation.

PC1 was significant for treatment, while PC2 was significant for the varietal effect (Table 2). Variation in PC1 was mostly contributed to by the positive coefficients of main axis length at Pr4, leaf injury scores at the fourth leaf, main axis diameter, length of first-order lateral roots, the diameter of first-order lateral roots, the diameter of second-order lateral roots, and density of root hairs of first-order lateral roots (Table 2, Figure 9). Additionally, variation in PC1 between three high-yielding and three low-yielding wheat genotypes between control and PEG-induced osmotic stresses, as reflected in biplot, was largely contributed to by the negative coefficients of the length of seminal root, the total number of roots and phytomers per tiller, number of live leaves, length of seminal roots, chlorophyll content, and shoot dry weight, as well as the positive coefficients of main axis length at Pr4, length of first-order lateral roots, and the density of root hairs of first-order lateral roots. PC1 accounted for a greater separation of six wheat genotypes between PEG-treated condition and control condition for their positive and negative PC scores, respectively (Figure 9). By contrast, PC2 separated three high-yielding and osmotic-stress-tolerant wheat genotypes (BARI gom 24, 25, and 33) from the low-yielding genotypes (Kheri, Sonora, and Kalaysona) for their positive and negative PC scores (Figure 9). These high-yielding genotypes contributed to greater and positive coefficients for root dry weight, the number of roots and root-bearing phytomer per tiller, the diameter of second-order lateral roots, and length, diameter, and density of root hairs in PC2 (Figure 9). 

## 3. Discussion

In plants, the root system is the key organ that is involved in water uptake and nutrient acquisition to influence the growth, development, and yield of the plants [35,36]. Under water-deficient conditions or osmotic stress, plants modify their root systems to cope with the altered situation [16,24,25,26,27]. Only a few previous studies looked at the alteration of root systems at the phytomer level [24,26,27,34]. Here, we discussed the modifications of individual roots of wheat plants under PEG-induced osmotic stress at the phytomer level.

### 3.1. PEG Stress Reduced New Root Formation Activity but Increased Length of Root Axes 

A significant reduction in the total number of phytomers and the total number of roots per tiller under PEG-induced osmotic stress, compared to control, indicated that PEG stress significantly reduced new root formation activity under osmotic stress (Figure 4). The first visual symptom of PEG stress was senescing of live leaves and degradation of chlorophyll (Figure 2, [27]). A reduction in the number of live leaves per plant by 22% was associated also with a 30% reduction in shoot dry weight. The reduced number of live leaves and chlorophyll content in the unit leaf area probably reduced the photosynthetic capacity of the individual tillers, which is associated with the lower photoassimilate biosynthesis [37]. The reduction of new root formation was probably associated with reduced substrate availability at the youngest phytomer position [33]. In perennial ryegrass, modeling data indicated that plants produce new roots under high photosynthetic carbon availability [33]. By contrast, wheat plants under salinity stress increased new root formation and thus increased the total number of adventitious roots per tiller at 12 days after treatment [34]. This contrasting result might be associated with the nature of the stress environment affecting the physiological process of the plants. Under salinity stress, there was a 2.1- and 23.6-fold decrease in the length of root axes at root-bearing phytomers 1 and 2, respectively, after 12 days of stress indicating that salinity stress-triggered plants to produce new roots by compensating the growth of existing roots. In this study, root axis length in root-bearing phytomers 2–4, maximum main axis length, and length of first-order lateral roots increased by 11.7% and 102.7%, respectively, indicating that wheat plants under osmotic stress increased root lengths probably to explore deeper soil depth to uptake water for their survival. Wheat plants under dry soil environments produced large root systems with longer axes length to explore more soil horizons for uptaking water and nutrients [22]. 

### 3.2. PEG-Induced Osmotic Stress Altered the Morphology of Lateral Roots

PEG stress enhanced the growth of the main root axis and lateral roots and thus increased root dry weight (RDW) (Figure 4 and Figure 5). Increment in length, diameter, and density of second-order lateral roots in the genotypes BARI Gom-24 and BARI Gom-25 indicated their tolerance strategies under osmotic stress. An increase in root diameter under stressed conditions increases surface area to facilitate water uptake in tolerant plants [38]. On the other hand, reduction in length, diameter, and density of second-order lateral roots in the genotype Kalaysona indicated its susceptibility to osmotic stress. 

In fact, a decrease in root length might be associated with the drop of relative turgidity and dehydration of protoplasm, linked with a loss of turgor and reduced expansion and division of cells featuring as a coping mechanism to survive under water-stressed conditions [39,40]. Therefore, the enhanced growth of lateral roots by the self-triggered re-modeling of root architecture suggested that the plants might have concentrated to elongate their root system both vertically and horizontally to explore new soil horizons to uptake water and nutrients. This result also indicated that the high-yielding genotypes such as BARI Gom-24 and BARI Gom-25 might be able to maintain the turgor pressure under PEG-induced osmotic stress, which enabled them to elongate their roots to resist the stress. 

### 3.3. Effect of PEG Stress on Root Hairs

Root hairs are essential tools for nutrient acquisition in all growing conditions, and they have the greatest contribution toward the total root length and surface area [24]. The density of root hairs of the main axis in the BARI Gom-24 under PEG-treated condition increased by 22%, while it was reduced by 3% in the genotype Kalaysona (Table S1). When plants are exposed to drought stress, the lateral roots develop more root hairs [41]. The increase of root hair density at the main axis and lateral roots might influence the absorption of more water from the root zone to lessen the effect of osmotic stress. Plants use the plasticity of their root hairs to initially increase surface area to uptake water and nutrition under stressful conditions [17,21,42]. The wheat genotypes BARI Gom-24 and BARI Gom-25, in this study, possibly increased the root surface area by increasing the length and density of root hairs [34].

### 3.4. Density of Lateral Roots and Root Hairs, Length and Diameter of the Main Axis, and First-Order Lateral Roots Were Strongly Associated with the Treatment Effect

A negative association between leaves and root production, as indicated in PC1, suggested that shoot growth was much more sensitive, compared to root growth under osmotic stress, justifying the previous reports [43,44]. PC1 further designated the treatment effect on wheat genotypes by showing that density of lateral roots and root hairs, length and diameter of the main axis, and first-order lateral roots were strongly associated with the treatment effect (Figure 9). Positive coefficients of those traits showed a strong coherent relationship to each other and thus expressed a treatment effect in both tolerant and susceptible wheat genotypes. 

### 3.5. Root Dry Weight, Density of Root Hairs and Second-Order Lateral Roots, Length of the Main axis, and First-Order Lateral Roots Traits Separated High-Yielding and Low-Yielding Genotypes

PC2 represented the separation of high-yielding wheat genotypes from low-yielding wheat genotypes with a positive coefficient for root dry weight, main axis length of Pr4, length of first-order lateral roots, the density of second-order lateral roots, density of root hair of the main axis, length of root hair in first-order lateral roots, etc. (Figure 9). The proliferation of root hairs evidently increased the interacting root surface area between roots and its medium by maximizing water and mineral absorption [45,46,47,48]. These results also indicated that the high-yielding genotypes BARI Gom-24 and BARI Gom-25 possibly enhanced water absorption under osmotic stress, compared to susceptible genotypes, by increasing their root surface area through the proliferation of the growth of root hairs and lateral roots. 

## 4. Materials and Methods

### 4.1. Plant Culture and Management 

Plants were grown in a plant culture room (Figure S1). Seeds of 22 wheat genotypes were collected from Bangladesh Agricultural Research Institute (BARI) based on distinctive entities (Table 3). 

Seeds of each genotype were germinated in individual Petri dishes. After one week, seeds were grown into healthy, dark green seedlings, with proper rooting and shooting. Two treatments, 0% PEG (control) and 10% PEG (wv), were applied following a completely randomized design to test the effect of PEG-induced osmotic stress [24,34]. Seedlings with synchronized leaf appearance were grown hydroponically in the individual trays following Robin et al. [24,34]. All plants under study were under similar management. There were six replicates per genotype during transplanting seedlings. Polyethylene glycol 6000 (PEG-6000, Merck-Schuchardt, Hohenbrunn, Germany) was applied to introduce osmotic stress when the plants were 14 days old. The water potentials, as an indicator of the osmotic status of the nutrient solutions, were measured before and after adding PEG. The water potentials before adding PEG and that of control treatment ranged between 0.08 and 0.09 MPa. Root data were accounted for the individual plants, and therefore, each of the individual plants of each genotype under each treatment was considered as an experimental unit. The nutrient solution in the hydroponic culture was replaced every week. pH was adjusted between 5.8 and 6.0 [50]. Plants were cultured at 20 ± 2 °C temperature, at 250 ± 10 micromole m^−2^ s^−1^ PPFD (photosynthetic photon flux density) light intensity. The cool white fluorescent lamps were used as a right source for maintaining the 16:8 h day:night cycle. Proper air circulation in the hydroponic culture was maintained by using aquarium air pumps. The composition and concentration of the nutrients were as follows: 1 mM NH_4_NO_3_, 0.6 mM NaH_2_PO_4_·H_2_O, 0.6 mM MgCl_2_·H_2_O, 0.3 mM K_2_SO_4_, 0.3 mM CaCl_2_·H_2_O, 50 µM H_3_BO_3_, 90 µM Fe-EDTA, 9 µM MnSO_4_·4H_2_O, 0.7 µM ZnSO_4_·7H_2_O, 0.3 µM CuSO_4_·5H_2_O, and 0.1 µM NaMoO_4_·2H_2_O dissolved in water [24,34]. 

### 4.2. Measurements and Data Collection

**Figure 10:**
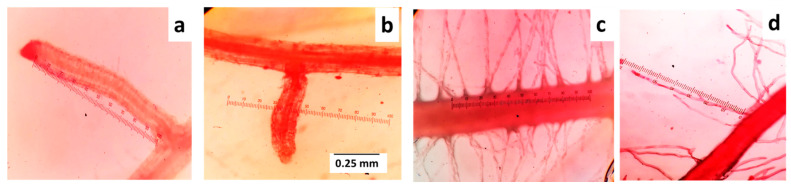
Measurements of root morphology and root hair traits: (**a**) length of second-order lateral roots; (**b**) diameter of second-order lateral roots; (**c**) density of root hairs of main axis; (**d**) length of a root hair.

Briefly, 14 days after applying 10% PEG-6000, plant height was measured with a 100 cm ruler, the number of total live leaves was counted, and chlorophyll content of all leaves was measured with the help of a chlorophyll meter (SPAD-502 PLUS, 3 V; 200 Mw; Konica Minolta Inc., Osaka, Japan) (Figure S1). Measurements related to individual root morphology and root hair traits at different root-bearing phytomers of individual plants were carried out. The destructive harvest was conducted at 31 days age of plants and 17 days after commencing PEG treatment [34,51]. Root measurements of the variables, as mentioned in Table 4, were carried out by following Robin et al. [34] (Figure 10). Root hairs were visualized in a 0.5% safranin solution, dissolved in 50% alcohol, to measure those at 100x magnification. The dry weights of the dissected shoots and roots of the individual tillers were recorded after drying those at 60 °C for 3 days. 

### 4.3. Statistical Analysis of Data 

The data obtained from this study were analyzed using Minitab 17 statistical software package (Minitab Inc., State College, PA, USA). The variations obtained due to treatments, genotypes, and treatment–genotypes were explored using a general linear model. Tukey’s pairwise comparison was deployed as a post hoc analysis to distinguish any significant differences among treatments, genotypes, and treatment–genotype interactions. A principal component analysis was carried out for the selected traits, and data for three high-yielding genotypes—BARI Gom-24, BARI Gom-25, and BARI Gom-33 (comparatively less-affected)—and three low-yielding genotypes—Kalaysona, Sonora, and Kheri (highly affected)—were collected, showing significant variations, to discover a pattern of association between resistant genotypes and root traits. A separate ANOVA was conducted to explore treatment and genotype effects in the PC scores. A biplot was constructed to visualize differences between high-yielding (resistant) and low-yielding (susceptible) genotypes associated with differences in treatment of the root traits. A Pearson correlation analysis was carried out with selected traits to explore relationships among them (Table S3).

## 5. Conclusions

Plants under osmotic stress showed significant alterations in root characteristics to survive under the PEG-induced osmotic stress environment. The density of lateral roots and root hairs, length and diameter of the main axis, and first-order lateral roots strongly altered under osmotic stress. Changes in the traits of lateral roots profoundly separated high-yielding genotypes from the low-yielding wheat genotypes. Data from this study can be used to construct a root ideotype, to model wheat genotypes for drought-prone environments, and also to exploit it in future breeding programs. 

## Figures and Tables

**Table 1:** Analysis of variance (mean squares) of different traits of 22 wheat genotypes (number of observations, n = 132 per treatment) under PEG-induced osmotic stress: PH = plant height (cm); TLL = total no. of live leaves; ChlC = chlorophyll content (SPAD value); LS4 = leaf injury scores for the fourth leaf; LS5 = leaf injury scores for the fifth leaf; SDW = shoot dry weight (g); RDW = root dry weight (g); TPr = total number of phytomers per tiller; TR = total no. of roots per tiller; NSR = no. of seminal roots; LSR = length of seminal roots (cm); MALmax = maximum main axis length (cm); MALPr1–Pr4 = main root axis length at phytomer 1–4 (cm); MAD = main axis diameter (mm); PAL = length of first-order lateral roots (cm); PAD = diameter of first-order lateral roots (mm); DPA = density of first-order lateral roots (no. mm^−1^); SAL = length of second-order lateral roots (cm); SAD = diameter of second-order lateral roots (mm); DSA = density of second-order lateral roots (no. mm^−1^); DRHMA = density of root hairs of main axis; DRHSA = density of root hairs of second-order lateral roots (no. mm^−1^); DRHPA = density of root hairs of first-order lateral roots (no. mm^−1^); RHLPA = length of root hairs of first-order lateral roots (µm).

Sources of Variation	Traits				
	**PH**	**TLL**	**ChlC**	**LS4**	**LS5**
Treatment (T)	1075.6 ***	25.5 ***	138.1 ***	8.8 ***	35.0 ***
Genotype (G)	135.7 ***	1.1 **	36.2 ***	3.5 ***	4.7 ***
T × G	35.4 **	0.5	15.4	2.9 ***	2.3 **
	**SDW**	**RDW**	**TPr**	**TR**	**NSR**
Treatment (T)	0.59 ***	0.14 ***	45.9 ***	90.01 ***	26.67 ***
Genotype (G)	0.045 **	0.002 ***	1.19	12.27 ***	1.69 **
T × G	0.015 *	0.0007 ***	1.43 *	7.25 ***	1.705 **
	**LSR**	**MALPr1**	**MALPr2**	**MALPr3**	**MALPr4**
Treatment (T)	46.80 ***	2.13 **	639.1 ***	408.4 **	1534.7 **
Genotype (G)	13.63 ***	0.72 **	135.12 **	149.2 **	169.9 ^NS^
T × G	7.58 **	0.78 ***	118.2 *	91.9 ^NS^	138.7 ^NS^
	**MALmax**	**MAD**	**PAL**	**PAD**	**DPA**
Treatment (T)	669.06 ***	2.13 ***	244.309 ***	0.63 ***	280.98 ***
Genotype (G)	96.59 **	0.038 ***	6.02 ***	0.014 ***	2.77 ***
T × G	82.16 *	0.065 ***	5.15 ***	0.01 ***	3.37 ***
	**SAL**	**SAD**	**DSA**	**DRH_MA_**	**DRH_SA_**
Treatment (T)	3.24 ***	0.005 **	331.26 ***	140.01 ***	1256.64 ***
Genotype (G)	0.28 ***	0.003 ***	11.83 ***	17.14 ***	12.98 ***
T × G	0.51 ***	0.003 ***	6.93 ***	12.87 ***	9.79 ***
	**DRH_PA_**	**RHL_PA_**			
Treatment (T)	238.36 ***	380557 ***			
Genotype (G)	16.42 ***	93217 ***			
T × G	7.62 ***	69821 ***			

*, **, and *** = significant at ≤5%, ≤1%, and ≤0.1% levels of probability, respectively. NS, not significant.

**Table 2:** Coefficients of principal components for different morphological traits of 22 wheat genotypes under control and 10% PEG-induced hydroponic culture.

Traits	PC1	PC2	PC3	PC4
Plant height	−0.203	0.114	0.227	0.227
Number of live leaves	−0.270	0.042	−0.153	0.155
Chlorophyll content	−0.265	0.153	0.183	0.135
Leaf injury scores for the fourth leaf	0.217	−0.133	−0.279	0.304
Leaf injury scores at the fifth leaf	0.178	−0.179	−0.116	0.322
Shoot dry weight	−0.235	0.021	0.009	0.303
Root dry weight	−0.025	0.406	−0.173	0.103
Maximum main axis length	0.239	0.123	−0.114	0.275
Main axis diameter	0.213	0.291	−0.115	0.1
Length of first-order lateral roots	0.283	0.097	−0.021	−0.092
Diameter of first-order lateral roots	0.26	0.002	0.268	0.023
Density of first-order lateral roots	0.059	0.182	0.365	−0.061
Length of second-order lateral roots	−0.055	0.2	0.088	−0.297
Diameter of second-order lateral roots	0.207	0.321	0.128	−0.079
Density of second-order lateral roots	0.058	0.173	0.372	0.208
Length of seminal roots	−0.313	0.163	−0.057	0.121
Number of phytomers per tiller	−0.305	0.227	−0.102	0.029
Number of roots	0.09	0.259	−0.367	−0.187
Number of seminal roots	−0.257	0.194	−0.077	0.095
Main root axis length at phytomer 1	−0.032	0.012	−0.382	0.176
Root hair density on main axis	0.135	0.287	0.182	0.33
Density of root hairs on second-order lateral roots	0.148	0.253	−0.098	−0.206
Density of root hairs on first-order lateral roots	0.265	0.137	−0.036	0.259
Length of root hairs on first-order lateral roots	−0.037	0.289	−0.178	−0.253
**P(treatment)**	<0.001	0.425	0.853	0.728
**P(genotype)**	0.944	0.010	0.583	0.205
**% Variation explained**	32.2	17.4	11.9	9.2
**Eigenvalue**	7.73	4.17	2.85	2.22

**Table 3:** List of wheat genotypes used in this study, along with their notable characteristics (BARI, http://baritechnology.org/en/home/tech_commodity#result (accessed on 21 May 2021)).

Sl. No	Genotype	Given Identity	Year Released	Yield (t ha^−1^)	Characteristics
1	Durum	V1	Tetra-ploid wheat	—	—
2	BARI Gom-19 (Sourav)	V2	1998	3.5–4.5	Moderately heat tolerant
3	BARI Gom-20 (Gourab)	V3	1998	3.6–4.8	Heat sensitive
4	Sonalika *	V4	1974	3.0–3.5	Low-yielding variety
5	Kanchan	V5	1983	3.5–4.5	Low-yielding variety
6	Sonora-64	V6	1974	1.6–2.2	Low-yielding variety
7	Kalaysona	V7	1974	2.6–3.2	Low-yielding variety
8	Triticale	V8	Hexa-ploid	—	—
9	Kheri *	V9	Indigenous cultivar	2.0–2.5	Low yielding variety
10	BARI Gom-21 (Shatabdi)	V10	2000	3.6–5.0	Good level of tolerance to terminal heat stress
11	BARI Gom-22 (Sufi)	V11	2005	3.6–5.0	Tolerant to late heat stress
12	BARI Gom-23 (Bijoy)	V12	2005	4.3–5.0	Moderately heat tolerant
13	BARI Gom-24 (Prodip)	V13	2005	4.3–5.1	High-yielding, but heat sensitive
14	BARI Gom-25	V14	2010	3.6–5.0	Moderate level of tolerance to heat stress
15	BARI Gom-26	V15	2010	3.6–5.0	Tolerant to terminal heat stress in late seeding
16	BARI Gom-27	V16	2012	4.0–5.4	Moderate level of tolerance to heat stress
17	BARI Gom-28	V17	2012	4.0–5.5	Tolerant to terminal heat stress in late seeding
18	BARI Gom-29	V18	2014	4.0–5.0	Moderately tolerant to terminal heat stress
19	BARI Gom-30	V19	2014	4.5–5.5	Resistant to stem rust race, Ug 99 and leaf rust and moderately resistant to Bipolaris leaf blight disease
20	BARI Gom-31	V20	2017	4.5–5.0	Resistant to leaf rust and moderately resistant to Bipolaris leaf blight disease
21	BARI Gom-32	V21	2017	4.6–5.0	High yielding, early in maturity and tolerant to terminal heat stress, leaf rust, and tolerant to Bipolaris leaf blight disease
22	BARI Gom-33	V22	2017	4.0–5.0	Zn enriched (55–60 ppm) and tolerant to wheat blast disease

Source: Ghosh et al. [49].

**Table 4:** Measurements related to individual root traits and root hair traits of 22 wheat genotypes at 31 days age of plants and 17 days after commencing PEG treatment at different root-bearing phytomers. The youngest root-bearing phytomer (Pr) was considered as reference point and was considered as Pr1.

Types of Variable	Traits	Number of Data Recorded per Genotype	Data Availability (Supplementary Data File)	Unit
Number of roots	Number of seminal roots (NSR)	2	File S1	no.
	Total number of roots (TR) per tiller	2	File S1	no.
	Total number of phytomer (TPr) per tiller	2	File S1	no.
Length	Main axis length at the root-bearing phytomer position (Pr) 1 and 4	2	File S1	cm
	Length of first-order lateral roots (PAL)	10	File S1	cm
	length of second-order lateral roots (SAL)	10	File S1	cm
	Length of seminal roots (LSR)	10	File S1	cm
Diameter	Main axis diameter (MAD)	10	File S1	mm
	Diameter of first-order lateral roots (PAD)	10	File S1	mm
	Diameter of second-order lateral roots (SAD)	10	File S1	mm
Density	Density of first-order lateral roots (DPA)	10	File S1	no. mm^−1^
	Density of second-order lateral roots (DSA)	10	File S1	no. mm^−1^
Root hair traits	Density of root hairs of main axis (DRH_MA_)	5	File S2	no. mm^−1^
	Density of root hairs of second-order lateral roots (DRH_SA_)	5	File S2	no. mm^−1^
	Density of root hairs of first-order lateral roots (DRH_PA_)	5	File S2	no. mm^−1^
	Length of root hairs of first-order lateral roots (RHL_PA_)	5	File S2	µm

## Data Availability

All data included as supplementary materials.

## Supplementary Materials

The following are available online at https://www.mdpi.com/article/10.3390/plants10061042/s1, Figure S1: Culture of wheat genotypes in a plant culture room (a) measurement of chlorophyll content (b) root growth (c) inspection of oxygen supply and adding of nutrients (d) plants of twenty-two wheat genotypes before destructive harvest., Table S1: Comparison of means among wheat varieties, Table S2: Treatment × Variety interactions in shoot, root and root hair traits of 22 wheat variety under two treatments, Table S3: Correlation coefficients of root traits.
