# Supplementary material for: PEG-Induced Osmotic Stress Alters Root Morphology and Root Hair Traits in Wheat Genotypes
Source: Plants (Basel). 2021 May 21;10(6):1042. doi: 10.3390/plants10061042 (PMC8224394; doi:10.3390/plants10061042)
Supplement: Supplementary file 1 [file plants-10-01042-s001.zip › plants-1208959-supplementary/Supplementary materials- 17 April 2021.pdf]

Supplementary Data

Table S1: Comparison of means among wheat varieties. PH=Plant height (cm), TLL=Total no. of live leaves, ChlC= Chlorophyll content (SPAD value), LS4= **Leaf injury scores at 4th** leaf**, LS5=** Leaf injury scores at 5th leaf, SDW=Shoot dry weight (g), RDW=Root dry weight (g), TPr=Total number of phytomers per tiller, TR=Total no. of roots per tiller, NSR=No. of seminal roots, LSR=Length of seminal roots (cm), MAL=Main axis length at Pr4 (cm), MALPr1=Main root axis length at phytomer 1 (cm), MAD=Main axis diameter (mm), PAL=Length of first order lateral roots (cm), PAD=Diameter of first order lateral roots (mm), DPA=Density of first order lateral roots (no. mm^{-1}), **SAL=Length of second order lateral roots** (cm), SAD=Diameter of second order lateral roots (mm), DSA=Density of second order lateral roots (no. mm^{-1}), DRH_{MA}= Density of root hairs of main axis, DRH_{SA}= Density of root hairs of second order lateral roots (no. mm^{-1}), DRH_{PA}=Density of root hairs of first order lateral roots (no. mm^{-1}), RHL_{PA}=Length of root hairs of first order lateral roots (µm).

VARIETY	PH	TLL	Chl3	LS4	LS5	SDW	RDW	TPr	TR	NSR	LSR	MALPr4
Durum	40.33	5.00	31.25	3.00	4.33	0.35	0.06	6.50	9.00	4.50	2.36	49.00
Sourav	37.33	4.33	28.37	2.67	4.33	0.40	0.06	7.00	9.25	5.00	1.38	46.05
Gourab	41.35	4.00	27.23	3.33	6.00	0.35	0.05	6.75	8.75	5.50	2.30	55.88
Sonalika	40.45	4.17	29.22	4.33	6.33	0.39	0.06	7.00	7.50	4.50	2.68	46.68
Kanchan	34.08	4.33	28.85	3.33	4.33	0.33	0.04	6.00	7.50	5.50	2.40	53.25
Sonora-64	32.75	3.50	26.65	3.67	5.33	0.21	0.03	5.50	6.50	5.00	2.38	47.75
Kalaysona	32.00	3.83	25.50	6.67	8.67	0.40	0.03	5.50	5.00	6.25	1.78	48.50
Triticale	49.67	4.83	36.10	3.33	6.33	0.41	0.05	6.00	6.25	4.00	1.95	49.38
Kheri	46.00	3.67	27.03	5.00	6.67	0.34	0.03	6.25	7.00	5.25	4.15	42.63
BARI-21	38.42	4.33	25.03	3.33	4.33	0.44	0.06	6.50	8.25	4.50	2.30	55.13
BARI-22	49.83	4.83	28.82	5.00	6.33	0.56	0.07	7.00	8.00	4.75	3.28	56.38
BARI-23	37.37	4.33	25.90	5.00	7.00	0.37	0.06	6.25	9.50	5.00	3.06	44.50
BARI-24	43.17	4.17	30.48	4.33	5.67	0.43	0.10	7.75	12.25	5.75	1.94	58.85
BARI-25	35.55	4.33	28.82	5.00	6.00	0.35	0.09	7.00	9.00	6.25	3.50	59.75
BARI-26	37.17	3.83	28.32	4.00	5.67	0.29	0.03	5.75	8.75	4.25	4.01	47.63
BARI-27	35.83	4.00	28.87	4.67	6.67	0.25	0.08	5.50	5.75	5.25	3.21	44.85
BARI-28	40.17	4.67	30.30	3.67	6.00	0.38	0.06	6.50	7.75	6.00	4.06	56.80
BARI-29	42.33	4.67	31.72	4.33	6.00	0.48	0.07	6.50	7.00	4.25	2.87	47.88
BARI-30	37.67	4.50	26.45	4.00	6.00	0.44	0.08	6.50	10.75	4.75	4.70	47.38
BARI-31	37.50	4.00	28.58	4.67	7.00	0.34	0.05	6.50	9.50	5.25	2.36	46.25
BARI-32	39.17	5.00	30.80	4.33	5.67	0.50	0.07	6.75	11.00	5.00	3.60	53.55
BARI-33	43.00	4.67	28.95	4.33	7.33	0.56	0.07	6.75	9.00	4.25	3.28	50.60
**Mean**	**39.60**	**4.32**	**28.78**	**4.18**	**6.00**	**0.39**	**0.06**	**6.44**	**8.33**	**5.03**	**2.89**	**50.39**
**SEM**	**0.592**	**0.075**	**0.283**	**0.125**	**0.159**	**0.014**	**0.008**	**0.135**	**0.325**	**0.148**	**0.189**	**0.935**
**p value**	**<0.001**	**0.002**	**<0.001**	**<0.001**	**<0.001**	**<0.001**	**<0.001**	**0.115**	**<0.001**	**0.003**	**<0.001**	**0.005**

Table S1: Comparison of means among wheat varieties (Cont.)

VARIETY	MALP1	MAD	PAL	PAD	DPA	SAL	SAD	DSA	DRH_{MA}	DRH_{SA}	DRH_{PA}	RHL_{PA}
Durum	2.30	0.46	2.70	0.23	5.10	0.35	0.14	3.30	8.20	7.10	7.60	343.00
Sourav	2.63	0.37	2.99	0.22	5.80	0.46	0.15	3.90	6.00	8.50	8.30	579.00
Gourab	2.00	0.44	1.78	0.21	6.75	0.52	0.18	4.20	7.60	9.10	7.00	440.00
Sonalika	2.23	0.40	1.86	0.23	5.30	0.64	0.15	4.50	7.30	7.30	6.80	288.00
Kanchan	2.20	0.38	3.52	0.21	5.90	0.58	0.14	4.15	7.70	7.40	9.30	432.00
Sonora-64	1.78	0.39	1.64	0.25	5.90	0.54	0.15	4.50	6.90	8.00	7.70	548.00
Kalaysona	1.58	0.49	1.65	0.23	5.46	0.29	0.13	3.00	6.50	5.67	9.10	346.00
Triticale	2.10	0.51	2.06	0.24	5.65	0.52	0.14	3.60	6.70	4.73	5.80	376.00
Kheri	2.48	0.40	1.87	0.22	6.25	0.37	0.16	3.80	8.80	9.60	7.80	477.00
BARI-21	2.35	0.41	1.92	0.17	5.75	0.48	0.15	3.45	8.90	6.50	7.80	426.00
BARI-22	2.18	0.41	2.31	0.21	5.85	0.43	0.15	3.95	6.00	7.10	9.70	505.00
BARI-23	1.30	0.46	2.85	0.28	5.45	0.44	0.15	3.30	9.00	7.00	9.30	508.00
BARI-24	3.05	0.49	2.52	0.25	5.40	0.44	0.17	4.35	7.10	7.80	8.10	625.00
BARI-25	2.35	0.43	2.73	0.22	5.45	0.61	0.15	5.10	10.90	9.40	9.90	518.00
BARI-26	2.13	0.36	2.37	0.20	5.50	0.37	0.15	4.50	6.80	8.90	8.00	415.00
BARI-27	1.35	0.42	2.92	0.20	6.18	0.58	0.14	4.91	5.88	7.70	5.90	300.00
BARI-28	2.50	0.41	2.05	0.26	5.90	0.37	0.15	5.85	8.60	7.90	9.50	461.00
BARI-29	2.35	0.50	1.70	0.26	6.25	0.42	0.15	5.85	5.38	7.40	7.40	443.00
BARI-30	2.83	0.50	1.75	0.22	6.05	0.41	0.16	4.05	6.60	8.40	7.50	439.00
BARI-31	1.98	0.39	2.85	0.26	5.68	0.83	0.16	5.30	6.70	6.90	7.90	481.00
BARI-32	2.38	0.42	1.40	0.20	5.88	0.38	0.16	3.40	6.40	7.50	10.80	588.00
BARI-33	2.33	0.45	1.96	0.19	5.55	0.50	0.12	4.40	8.40	6.30	7.10	627.00
**Mean**	**2.20**	**0.43**	**2.24**	**0.23**	**5.77**	**0.48**	**0.15**	**4.24**	**7.38**	**7.55**	**8.10**	**462.05**
**SEM**	**0.096**	**0.007**	**0.273**	**0.0036**	**0.07**	**0.019**	**0.0014**	**0.078**	**0.166**	**0.156**	**0.157**	**10.76**
**p value**	**0.003**	**<0.001**	**<0.001**	**<0.001**	**<0.001**	**<0.001**	**<0.001**	**<0.001**	**<0.001**	**<0.001**	**<0.001**	**<0.001**

Table S2: Treatment × Variety interactions in shoot, root and root hair traits of 22 wheat variety under two treatments. PH=Plant height, TLL=Total no. of live leaves, LS4= Leaf injury scores at 4th leaves**,** **LS5=** Leaf injury scores at 5th leaves**,** ChlC= Chlorophyll content, SDW=Shoot dry weight, TPr=Total number of root bearing phytomers per tiller, NSR=Number of seminal roots, LSR=Length of seminal roots, MALPr1-Pr4= Main axis length at phytomers 1-4.

**VARIETY**	**PH**		**TLL**		**Chl3**		**LS4**		**LS5**		**SDW**		**TPr**		**NSR**	
	0%	10% PEG	0%	10% PEG	0%	10% PEG	0%	10% PEG	0%	10% PEG	0%	10% PEG	0%	10% PEG	0%	10% PEG
Durum	43.00	37.67	5.67	4.33	35.10	27.40	2.33	3.67	3.67	5.00	0.41	0.29	7.0	6.0	4.5	4.5
Sourav	41.00	33.67	4.33	4.33	28.37	28.37	**2.33**	3.00	**3.00**	5.67	0.49	0.31	7.5	6.5	5.5	4.5
Gourab	44.37	38.33	4.67	3.33	28.37	26.10	3.00	3.67	4.33	7.67	0.41	0.28	7.5	6.0	6.5	4.5
Sonalika	44.23	36.67	4.00	4.33	29.83	28.60	3.67	5.00	5.67	7.00	0.51	0.26	7.0	7.0	5.5	3.5
Kanchan	42.50	**25.67**	4.67	4.00	30.63	27.07	3.00	3.67	3.67	5.00	0.42	0.23	6.0	6.0	6.5	4.5
Sonora-64	34.50	31.00	3.67	3.33	31.60	**21.70**	3.00	4.33	4.33	6.33	0.25	**0.17**	6.5	4.5	4.5	5.5
Kalaysona	35.33	28.67	4.00	3.67	28.10	22.90	4.33	9.00	8.33	9.00	0.60	0.21	7.0	4.0	6.0	6.5
Triticale	53.33	46.00	5.33	4.33	36.27	35.93	3.00	3.67	5.00	7.67	0.42	0.41	7.0	5.0	4.5	3.5
Kheri	43.00	49.00	4.00	3.33	26.20	27.87	4.33	5.67	6.33	7.00	0.38	0.30	6.5	6.0	5.0	5.5
BARI-21	44.17	32.67	4.67	4.00	26.37	23.70	3.67	3.00	5.00	3.67	0.56	0.32	7.0	6.0	5.5	3.5
BARI-22	52.67	47.00	5.67	4.00	27.83	29.80	5.00	5.00	5.00	7.67	0.68	0.45	7.5	6.5	5.5	4.0
BARI-23	41.73	33.00	5.00	3.67	28.57	23.23	3.67	6.33	5.00	9.00	0.46	0.28	7.5	5.0	7.0	3.0
BARI-24	44.33	42.00	5.00	**3.33**	31.33	29.63	3.67	5.00	5.67	5.67	0.47	0.39	10.0	5.5	7.0	4.5
BARI-25	38.77	32.33	4.67	4.00	30.27	27.37	5.00	5.00	5.00	7.00	0.42	0.28	8.5	5.5	7.0	5.5
BARI-26	36.00	38.33	4.00	3.67	27.80	28.83	4.33	3.67	5.67	5.67	0.23	0.35	6.0	5.5	4.5	4.0
BARI-27	38.33	33.33	4.33	3.67	28.13	29.60	3.67	5.67	5.00	8.33	0.26	0.25	7.0	**4.0**	6.5	4.0
BARI-28	45.33	35.00	5.00	4.33	29.73	30.87	3.00	4.33	5.00	7.00	0.53	0.24	7.5	5.5	5.5	6.5
BARI-29	46.00	38.67	5.33	4.00	33.07	30.37	3.00	5.67	4.33	7.67	0.56	0.40	6.5	6.5	5.5	**3.0**
BARI-30	36.33	39.00	5.00	4.00	25.23	27.67	3.67	4.33	5.00	7.00	0.47	0.40	7.0	6.0	4.5	5.0
BARI-31	39.67	35.33	4.33	3.67	30.33	26.83	5.00	4.33	5.67	8.33	0.38	0.29	6.5	6.5	6.0	4.5
BARI-32	43.00	35.33	5.67	4.33	32.90	28.70	3.67	5.00	4.33	7.00	0.58	0.42	8.0	5.5	5.5	4.5
BARI-33	46.33	39.67	5.67	3.67	29.70	28.20	4.33	4.33	6.33	8.33	0.62	0.51	7.0	6.5	4.5	4.0
Mean	42.5	36.7	4.76	3.88	29.8	27.7	3.67	4.70	5.06	6.94	0.46	0.32	7.18	5.70	5.59	4.48
SEM	0.73	0.79	0.10	0.08	0.41	0.37	0.13	0.19	0.19	0.19	0.02	0.02	0.16	0.16	0.21	0.20
p value	0.002		0.307		0.057		0.003		0.03		0.027		0.044		0.003	

Table S2: Treatment × Variety interactions in shoot, root and root hair traits of 22 wheat variety under two treatments (cont.)

VARIETY	LSR		MALPr1		MALPr2		MALPr3		MALPr4	
	0%	10% PEG	0%	10% PEG	0%	10% PEG	0%	10% PEG	0%	10% PEG
Durum	2.3	2.44	1.80	3.30	5.85	21.55	10.85	24.30	21.45	31.00
Sourav	1.4	**1.32**	3.35	1.90	4.90	3.85	5.70	13.45	31.00	22.35
Gourab	1.9	2.72	2.15	3.50	4.90	5.30	9.50	19.70	15.60	26.50
Sonalika	1.8	3.53	2.20	2.75	7.90	6.45	10.15	17.40	16.05	32.15
Kanchan	1.6	3.20	2.55	1.85	7.15	18.50	17.30	30.00	44.25	38.85
Sonora-64	2.3	2.47	2.35	1.10	3.70	17.50	13.60	21.25	25.05	41.50
Kalaysona	2.0	1.55	1.50	1.65	3.15	11.50	29.00	13.00	43.00	49.00
Triticale	1.8	2.14	2.85	1.85	10.65	3.80	15.40	24.20	18.60	32.25
Kheri	3.1	5.16	3.10	2.35	14.25	9.30	19.60	12.70	26.50	26.50
BARI-21	2.6	2.02	2.90	1.80	6.30	19.50	18.00	32.00	27.25	35.75
BARI-22	2.2	4.36	2.85	2.00	15.50	21.20	16.05	28.75	23.50	33.00
BARI-23	1.5	4.64	2.45	1.15	9.35	4.30	14.75	10.90	18.75	17.30
BARI-24	2.1	1.79	4.25	2.75	7.40	4.80	11.60	10.05	18.00	22.25
BARI-25	2.9	4.07	3.35	1.35	7.95	22.05	8.85	14.90	12.20	40.75
BARI-26	2.8	5.18	2.25	2.00	3.85	5.35	7.45	21.25	27.00	32.00
BARI-27	4.9	1.55	0.95	1.50	2.90	11.00	4.90	20.00	9.70	33.00
BARI-28	3.5	4.60	2.25	2.75	3.70	19.00	12.95	22.50	20.05	57.50
BARI-29	3.1	2.62	2.40	2.80	19.25	26.75	33.00	36.00	35.50	45.75
BARI-30	4.3	5.07	3.70	1.95	12.80	5.35	25.50	10.55	28.00	32.35
BARI-31	2.4	2.36	1.60	2.35	3.25	3.45	5.75	13.50	23.50	37.50
BARI-32	2.5	4.76	3.85	1.90	6.70	3.25	12.80	5.60	19.75	19.60
BARI-33	3.0	3.53	2.50	2.15	6.55	7.75	23.50	13.00	25.50	26.25
**Mean**	2.55	3.23	2.60	2.12	7.63	11.43	14.83	18.86	24.10	33.32
**SEM**	0.205	0.325	0.10	1.25	0.85	1.47	1.01	1.50	1.41	2.04
**p value**	0.01		0.003		0.001		0.019		0.002	

Table S3: Correlation coefficients of root traits. PH=Plant height, TLL=Total no. of live leaves, LS4= **Leaf injury scores at 4th leaves**, LS5= Leaf injury scores at 5th leaves**,** ChlC= Chlorophyll content, SDW=Shoot dry weight, RDW=Root dry weight, TPr=Total number

of root bearing phytomers per tiller, TR=Total number of roots per tiller, NSR=Number of seminal roots, LSR=Length of seminal roots, MALmax= Maximum main axis length, MAD=Main axis diameter, PAL=Length of first order lateral roots, PAD=Diameter of first order lateral roots, DPA=Density of first order lateral roots, SAL=Length of second order lateral roots, SAD=Diameter of second order lateral roots, DSA=Density of second order lateral roots, DRHMA= Density of root hairs of main axis, DRHSA= Density of root hairs of second order lateral roots.

	RDW	TPr	TR	NSR	LSR	MALmax	MAD	PAL	PAD	DPA	SAL	SAD	DSA	DRHma	DRHsa
**TPr**	0.379NS														
**TR**	0.492NS	0.92***													
**NSR**	0.405NS	0.754**	0.867***												
**LSR**	-0.039NS	-0.012NS	-0.12 NS	-0.01 NS											
**MALmax**	0.664*	-0.101 NS	0.091 NS	0.008 NS	-0.278 NS										
**MAD**	0.34NS	-0.383 NS	-0.358 NS	-0.369 NS	0.011 NS	0.252 NS									
**PAL**	0.481NS	-0.381 NS	-0.149 NS	-0.034 NS	0.221 NS	0.606*	0.585*								
**PAD**	0.073NS	-0.612*	-0.528 NS	-0.461 NS	-0.042 NS	0.206 NS	0.719**	0.525 NS							
**DPA**	-0.145NS	-0.759**	-0.634*	-0.348 NS	0.341 NS	-0.051 NS	0.381 NS	0.545 NS	0.521NS						
**SAL**	0.162NS	-0.067 NS	-0.068 NS	-0.316 NS	0.42 NS	-0.002 NS	0.097 NS	0.217 NS	0.088 NS	0.303 NS					
**SAD**	0.257NS	0.309 NS	0.266 NS	0.169 NS	-0.019 NS	0.144 NS	-0.09 NS	-0.134 NS	0.232 NS	-0.176 NS	0.19 NS				
**DSA**	0.448NS	-0.337 NS	-0.262 NS	-0.347 NS	0.38 NS	0.378 NS	0.546 NS	0.588*	0.677*	0.373 NS	0.508 NS	0.251 NS			
**DRHma**	0.392NS	-0.032 NS	-0.113 NS	-0.008 NS	0.748**	0.01 NS	0.389 NS	0.497 NS	0.285 NS	0.321 NS	0.341 NS	-0.027 NS	0.638*		
**DRHsa**	0.205NS	-0.186 NS	-0.125 NS	0.005 NS	0.416 NS	0.503 NS	0.084 NS	0.533 NS	0.313 NS	0.205 NS	0.001 NS	0.26 NS	0.442 NS	0.392 NS	
**DRHpa**	0.334NS	-0.374 NS	-0.447 NS	-0.349 NS	0.203 NS	0.236 NS	0.691*	0.579*	0.591*	0.473 NS	0.238 NS	-0.01 NS	0.488 NS	0.658*	0.297 NS

*, **, and *** = Significant at ≤ 5%, ≤ 1%, and ≤ 0.1% levels of probability, respectively and NS= Non-significant

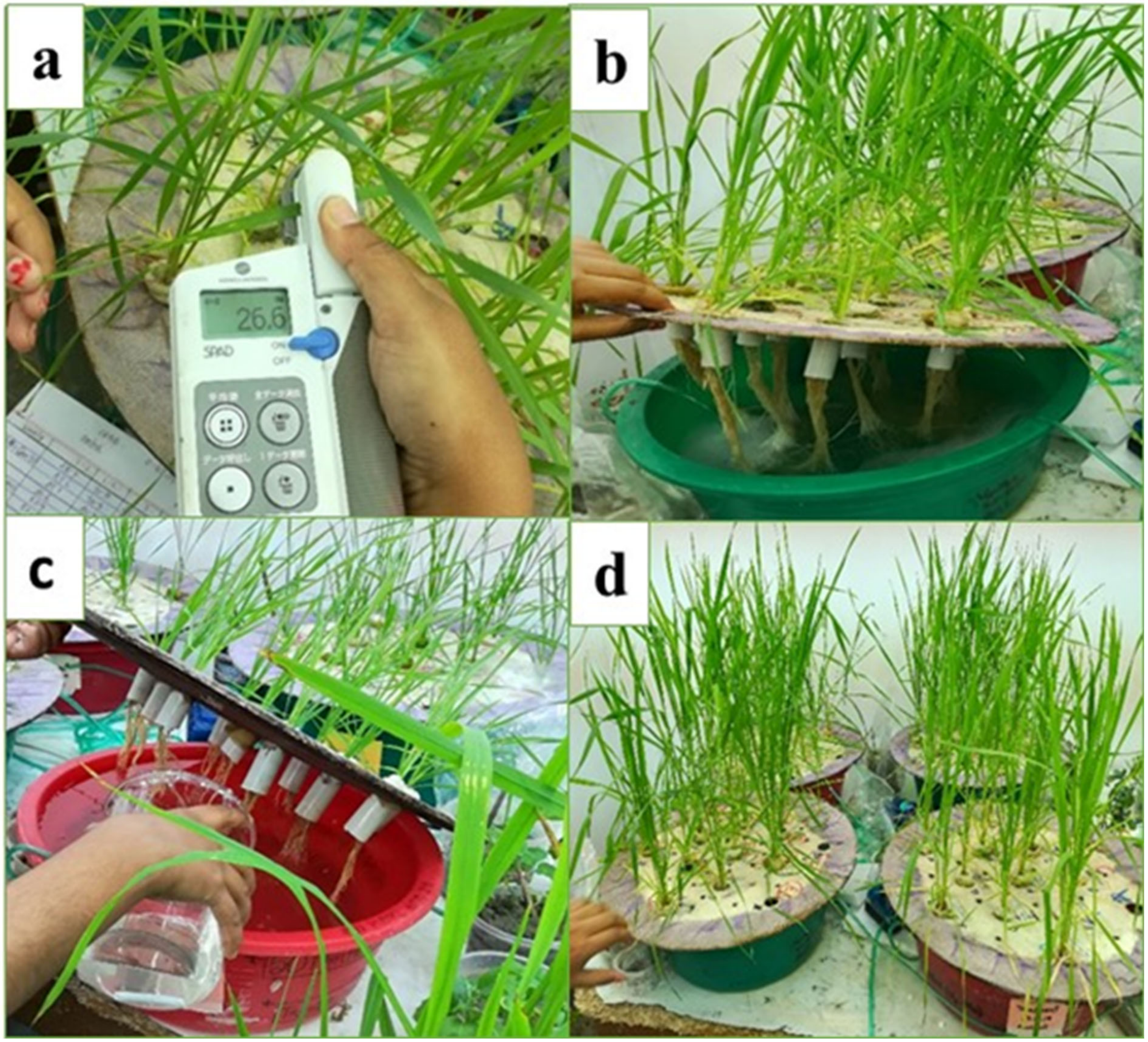

Figure S1: Culture of wheat genotypes in a plant culture room a) measurement of chlorophyll content b) root growth c) inspection of oxygen supply and adding of nutrients d) plants of twenty-two wheat genotypes before destructive harvest.
